# A Systematic Review of ABCB1 Polymorphisms and Antiseizure Medication Resistance: Insights from Effect Size and Study Power Analysis

**DOI:** 10.3390/ijms26125548

**Published:** 2025-06-10

**Authors:** Aurelija Daškevičiūtė, Edgaras Zaboras, Jonas Navalinskas, Karolis Baronas, Arminas Jasionis, Eglė Navickienė, Rūta Mameniškienė

**Affiliations:** 1 Clinic of Neurology and Neurosurgery, Institute of Clinical Medicine, Faculty of Medicine, Vilnius University, LT-08661 Vilnius, Lithuaniaruta.mameniskiene@mf.vu.lt (R.M.); 2 Faculty of Medicine, Vilnius University, LT-03101 Vilnius, Lithuania; edgaras.zaboras@mf.stud.vu.lt (E.Z.); jonas.navalinskas@mf.stud.vu.lt (J.N.); 3 Department of Human and Medical Genetics, Institute of Biomedical Sciences, Faculty of Medicine, Vilnius University, LT-08661 Vilnius, Lithuania; 4 Vilnius University Hospital Santaros Clinics, LT-08661 Vilnius, Lithuania

**Keywords:** drug-resistant epilepsy, ABCB1, antiseizure medications, single-nucleotide polymorphisms, rs1045642, rs2032582, rs1128503

## Abstract

The most investigated ABCB1 single-nucleotide polymorphisms (SNPs) related to antiseizure medication resistance are rs1045642 (c.3435C>T, p.Ile1145=), rs2032582 (c.2677G>T/A, p.Ala893Ser/Thr), and rs1128503 (c.1236C>T, p.Gly412=). We conducted a literature review to evaluate the genotype frequencies of rs1045642, rs2032582, and rs1128503 SNPs in different ancestries among the drug-resistant and drug-responsive epilepsy groups. Furthermore, we performed effect size and study power analyses and determined the expected sample size to reach a study power of 0.8 for each conducted research. High and very high statistical power for the rs1045642, rs2032582, and rs1128503 polymorphisms was achieved in 58.0, 60.7, and 31.8% of the studies, respectively. The effect sizes (ES) of rs1045642, rs2032582, and rs1128503 ranged from 0.03–1.04, 0.06–0.92, and 0.04–0.64, respectively. The required sample sizes for rs1045642, rs2032582, and rs1128503 ranged from 9–13,000, 12–2600, and 24–5700 participants, respectively. None of the polymorphisms showed a statistically significant association with antiseizure medication resistance in the forest plots. Our analysis provides valuable guidance for future genetic association studies in the field of drug-resistant epilepsy.

## 1. Introduction

The International League Against Epilepsy (ILAE) proposed a definition for drug-resistant epilepsy, which is the failure of adequate trials of two tolerated, appropriately chosen, and used antiseizure medication (ASM) schedules (whether as monotherapies or in combination) to achieve “sustained seizure freedom” [1]. Majorly, there were seven to eight ASMs to choose from until 1993; afterward, more than 19 new ASMs were approved [2]. The new ASMs have widened the treatment choices for clinicians; however, they have no significant impact on the outcomes of patients with epilepsy [3]. A recent meta-analysis showed that approximately 20% of patients with new-onset epilepsy would develop drug-resistant epilepsy (DRE) [4].

**Figure 1:**
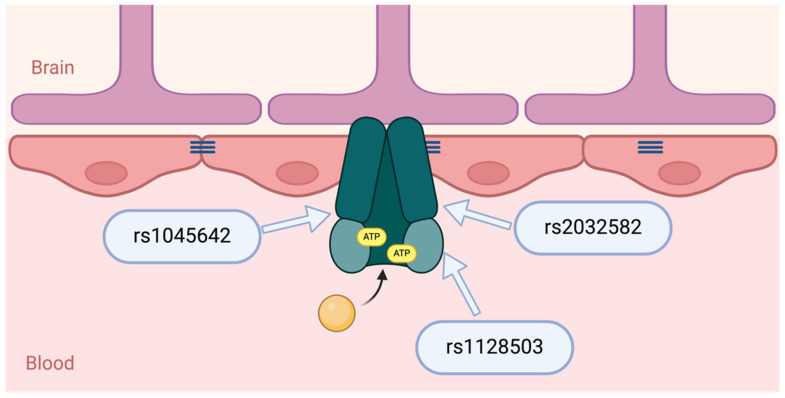
Schematic representation of the ABCB1 (P-glycoprotein) transporter at the blood–brain barrier and its association with key single-nucleotide polymorphisms (SNPs): rs1045642 (c.3435C>T, p.Ile1145=), rs2032582 (c.2677G>T/A, p.Ala893Ser/Thr), and rs1128503 (c.1236C>T, p.Gly412=). The indicated SNPs in the ABCB1 gene may affect P-gp function, potentially altering drug resistance in epilepsy. The orange circle represents a drug substrate transported by P-gp, and ATP molecules (yellow) indicate the energy source required for active efflux. Created in BioRender. Daškevičiūtė, A. (2025) https://BioRender.com/m75w336, accessed on 11 May 2025.

Several hypotheses have been proposed to explain drug resistance in epilepsy, including pharmacokinetics, neural networks, intrinsic severity, gene variants, targets, and transporters [5]. Among these, the efflux transporter gene variant hypothesis has been the most explored and cited. Several efflux transporters may be involved in the permeability of the blood–brain barrier to ASMs. They include ATP-binding cassette transporters, especially P-glycoprotein (P-gp, ATP-Binding Cassette Subfamily B Member 1 [ABCB1]), multidrug resistance-associated proteins (MRPs, ABCC), and breast cancer resistance proteins (BRCP, ABCG2) [6]. The most investigated ABCB1 single-nucleotide polymorphisms (SNPs) related to ASM resistance are rs1045642 (c.3435C>T, p.Ile1145=), rs2032582 (c.2677G>T/A, p.Ala893Ser/Thr), and rs1128503 (c.1236C>T, p.Gly412=) (Figure 1).

Studies evaluating the relationship between polymorphisms and resistance to ASMs yielded inconsistent results. Some investigators have found a statistically significant association between ABCB1 polymorphisms and ASM resistance, whereas others have failed to replicate these results, leading to an ongoing debate in this field. Failure to reproduce findings might result from several factors, such as heterogeneous epilepsy study groups, differences in allele and genotype frequencies between populations of different ancestries, and inadequate sample sizes and study power [7]. Therefore, performing sample size and study power calculations during the design stage of a study to detect meaningful associations is essential.

We aimed to conduct a literature review to evaluate the genotype frequencies of rs1045642 (c.3435C>T, p.Ile1145=), rs2032582 (2677T>G/A, Ser893Ala/Thr), and rs1128503 (c.1236C>T, p.Gly412=) in different ancestries among the drug-resistant and drug-responsive epilepsy groups. Furthermore, we performed effect size and study power analyses of existing studies and determined the expected sample size to reach a power of 0.8 for each study. These calculations helped to determine the effect size and sample size requirements and offered guidance for future genetic association studies in drug-resistant epilepsy. We also assessed the genotype frequencies of the included populations and conducted a forest plot analysis.

## 2. Materials and Methods

### 2.1. Information Sources and Search Strategy

We obtained publications from the National Center for Biotechnology Information Database of Single-Nucleotide Polymorphisms (dbSNP) that evaluated the relationship between rs1045642 (c.3435C>T, p.Ile1145=), rs2032582 (c.2677G>T/A, p.Ala893Ser/Thr), or rs1128503 (c.1236C>T, p.Gly412=) and drug-resistant epilepsy. Each polymorphism identifier was entered into the dbSNP search box. Subsequently, the section “publications” was accessed from the Reference Single-Nucleotide Polymorphism (SNP) report page. We included studies in this analysis if all the following criteria were met: (1) The study investigated the association of rs1045642, rs2032582, or rs1128503 polymorphisms with drug-resistant epilepsy (drug-resistant epilepsy was defined according to the criteria used by the original study authors), (2) the study provided the frequency of genotypes and alleles of both drug-resistant and drug-responsive epilepsy groups, (3) the study evaluated Hardy–Weinberg equilibrium (HWE), and (4) the study was published between 2003 and 2025. The exclusion criteria included the following: (1) The study investigated only the relationship between rs1045642, rs2032582, or rs1128503 polymorphisms and blood concentrations of ASMs, (2) the article was not written in English, (3) the full-text article was unavailable, and (4) the study was performed in vitro. Systematic reviews and meta-analyses were evaluated for studies that were not identified in the initial search and were added to our study as cross-references.

Additionally, we searched three more databases: Cochrane, Scopus, and Springer Nature Link. In the Cochrane database, the literature search was carried out using the following string: (ABCB1 OR C3435T OR rs1045642 OR G2677A OR rs2032582 OR C1236T OR rs1128503) AND (epilepsy OR drug resistant epilepsy). These search criteria gave a list of 5 studies, all of which met the exclusion criteria. The Scopus database was searched using the string “ABCB1 AND epilepsy”, which yielded 228 studies. After removing the duplicates from the initial dbSNP search and excluding studies based on the predetermined criteria, five additional studies were included in the analysis. In the Springer Nature Link database, the search using the terms “epilepsy AND ABCB1” returned 410 results. However, the majority of these articles were duplicates of previously identified studies or met one or more exclusion criteria and were therefore not included in the final analysis.

**Figure 2:**
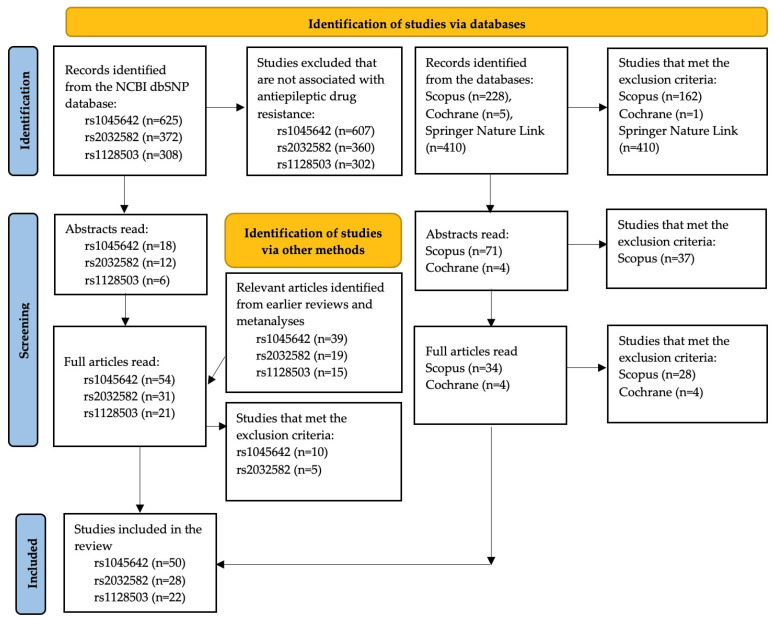
PRISMA 2020 flow diagram; 2020 statement: An updated guideline for reporting systematic reviews [8].

The selection process for publications meeting the inclusion criteria consisted of two stages. First, two reviewers independently screened the titles of all studies identified through the search to assess their eligibility. After the initial identification, abstracts were screened, and duplicate or ineligible articles were excluded. The remaining studies underwent a full-text review, which was independently conducted by two reviewers. Any discrepancies were resolved through discussion and consensus. Figure 2 shows a modified PRISMA flow diagram illustrating the selection process.

### 2.2. Data Extraction and Statistical Analysis

The following information was extracted to a data analysis sheet after the inclusion of the studies: the first author, publication year, country, total number of subjects, number of drug-resistant and drug-responsive subjects, frequency of genotypes in each group in absolute numbers, and, if available, the percentage frequency and *p*-values. If the percentage frequency was unavailable, we calculated it using the absolute frequency and transformed it into a decimal representation.

The G*Power 3.1 tool was used to conduct the study’s power and effect size calculations and to determine the required sample size to reach a 0.8 study power with a given effect size. We selected a study power threshold of 0.8, which is considered the standard in biomedical research, balancing the risks of Type I and II errors. Calculations were performed in two steps, which included the post hoc and a priori analyses. In the post hoc analysis, the chi-squared (χ^2^) test family and “goodness-of-fit tests: contingency tables” were selected. The decimal frequency of each genotype in the drug-resistant epilepsy group was entered into the frequency table p (H0) cells, and the decimal frequency of genotypes in the drug-responsive epilepsy group was entered into the p (H1) cells. We added 0.5 to every genotype’s absolute frequency if the genotype frequency was zero. The effect size was calculated and transferred to the main program window, where the total sample size of the study was entered. The significance level was set at 0.05. The degrees of freedom were computed as d.f. = (number of rows minus 1) × (number of columns minus 1). Thereafter, we used the G*Power 3.1 tool to calculate the actual power of the study. Subsequently, a priori analysis was performed, which included adjusting the study power to 0.8 and calculating the required sample size based on the effect size determined in the post hoc analysis. Forest plots were generated using the MetaGenyo tool (https://metagenyo.genyo.es/, accessed on 11 May 2025).

## 3. Results

### 3.1. rs1045642 (c.3435C>T, p.Ile1145=)

#### 3.1.1. Characteristics of Studies Included in the Analysis of rs1045642

**Figure 3:**
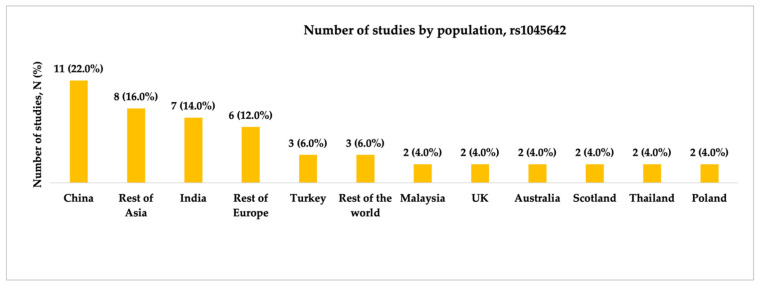
The number of included studies investigating the association between rs1045642 (c.3435C>T, p.Ile1145=) and antiseizure medication resistance by population. “Rest of Asia” includes Pakistan, Vietnam, Korea, Taiwan, Iran, Iraq, Jordan, and Japan; “Rest of Europe” includes Italy, Germany, Croatia, Macedonia, Spain, and Albania; “Rest of the World” includes Brazil, Tunisia, and Egypt.

**Figure 4:**
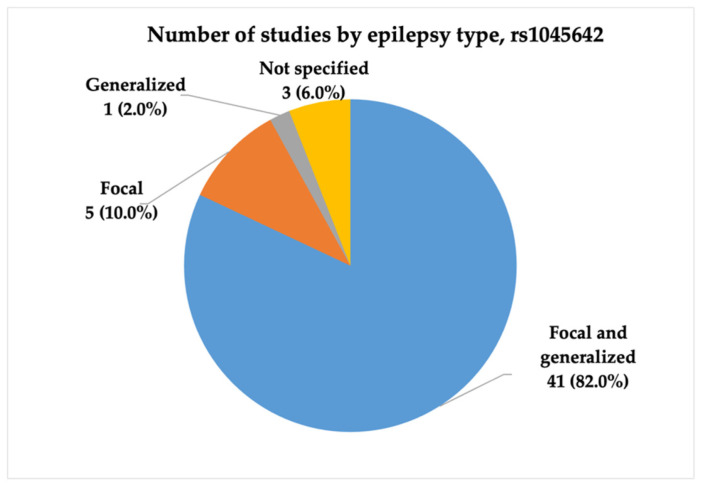
The distribution of included studies by epilepsy type for rs1045642 (c.3435C>T, p.Ile1145=). The majority of studies (82%) examined both focal and generalized epilepsy types, while smaller proportions focused exclusively on focal (10%) or generalized (2%) epilepsy.

**Figure 5:**
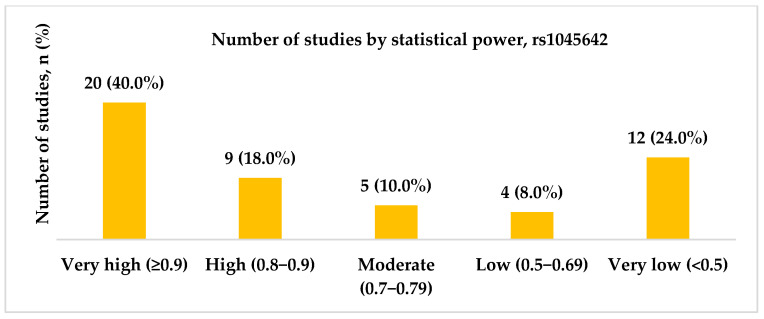
The distribution of included studies by statistical power for rs1045642 (c.3435C>T, p.Ile1145=). Most studies demonstrated either very high (≥0.9; n = 20) or very low (<0.5; n = 12) statistical power.

Fifty studies regarding the rs1045642 (c.3435C>T, p.Ile1145=) SNP were included in our final analysis. Studies were conducted between 2003 and 2024, with the highest number published in 2009 (N = 8, 15.7%). Most studies were conducted in China (including the Chinese Han population) (11, 22.0%), followed by India (7, 14.0%). Most studies (N = 41, 82.0%) included patients with both focal and generalized epilepsy, whereas five (10.0%) had focal epilepsy. The statistical power analysis revealed that 20 (40.0%) studies exhibited very high statistical power (1 − β ≥ 0.9), and nine studies (18.0%) achieved high statistical power (1 − β = 0.8–0.9). Figure 3, Figure 4 and Figure 5 present the distribution of included studies by population, epilepsy type, and statistical power, respectively.

#### 3.1.2. Genotype Associations, Effect Sizes, and Required Sample Sizes for rs1045642

Of the 20 studies with very high statistical power, the rs1045642 CC genotype was associated with ASM resistance in India [9], Thailand [9,10], Iran [11], Taiwan [12], and Pakistan [13]. However, the CC genotype has been associated with ASM responsiveness in the Han Chinese population [14]. The rs1045642 TT genotype was associated with ASM resistance in Malaysia [15], Tunisia [16], Japan [17], and Han Chinese [18,19], whereas the CT genotype was associated with ASM resistance in Thailand [20]. In the UK, the C allele was significantly overrepresented in the DRE group [21]. In Croatia, the analysis of the rs1045642 SNP in the G2677/C3435/C1236 haplotype revealed that the GG/CC/CC genotype combination was significantly overrepresented in patients with drug resistance [22]. Furthermore, distinct findings were observed in Turkey, where CC3435/GG2677 expression was considerably higher in the drug-responsive group [23]. Of the nine studies with high statistical power, the CC genotype showed a higher frequency in Iraqi patients [24].

The effect size for rs1045642 (c.3435C>T, p.Ile1145=) ranged from 0.03, requiring a calculated sample size of over 13,000 participants, to 1.04, detectable in only nine participants. The number of enrolled participants varied from 45 to 609 and 84 to 285 in the very high and high statistical power studies, respectively. Whereas in the low-power studies (Korea, Germany, China, and Macedonia), the sample size varied from 162–350 and would have needed a 33% to 100% increase to achieve a high-power study. In very low-power studies (Scotland, Korea, Ireland, Turkey, China, Brazil, India, and Albania), the number of participants ranged from 29–537. These studies would have required the sample sizes to be increased from 204 to more than 3000% to achieve a statistical power of 0.8. The genotype frequencies, effect sizes, and power analyses of the studies are presented in Table 1.

### 3.2. rs2032582 (c.2677G>T/A, p.Ala893Ser/Thr)

#### 3.2.1. Characteristics of Studies Included in the Analysis of rs2032582

**Figure 6:**
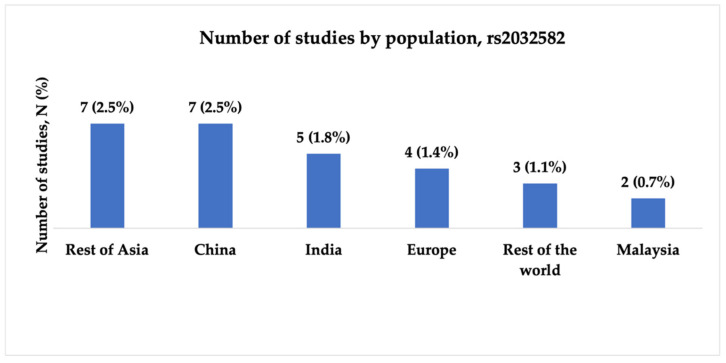
The number of included studies investigating the association between rs2032582 (c.2677G>T/A, p.Ala893Ser/Thr) and antiseizure medication resistance by population. “Rest of Asia” includes Korea, Japan, Taiwan, Pakistan, Jordan, Iran, and Turkey; “Europe” includes Germany, Spain, Croatia, and Poland; “Rest of the world” includes Brazil, Tunisia, and Egypt.

**Figure 7:**
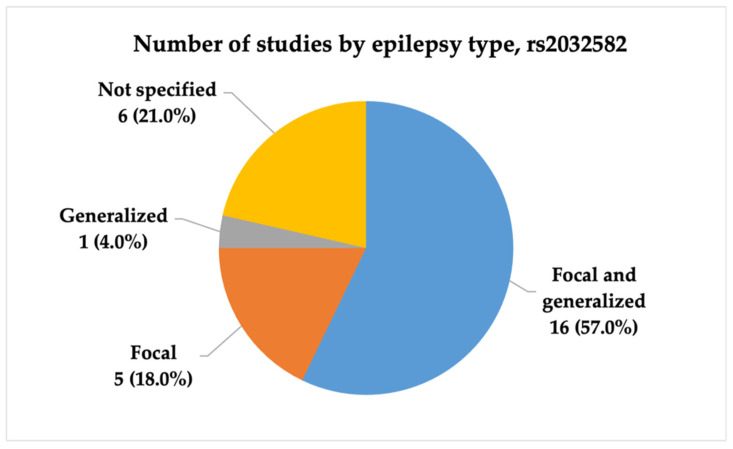
The distribution of included studies by epilepsy type for rs2032582 (c.2677G>T/A, p.Ala893Ser/Thr). The majority of studies (57%) included patients with both focal and generalized epilepsy, while fewer studies focused exclusively on focal (18%) or generalized (4%) epilepsy types.

**Figure 8:**
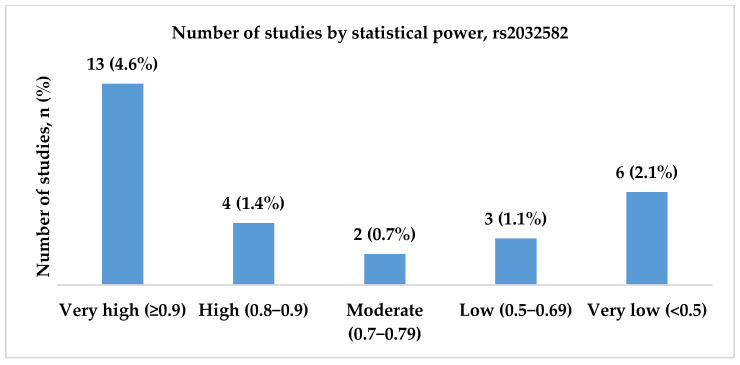
The distribution of included studies by statistical power for rs2032582 (c.2677G>T/A, p.Ala893Ser/Thr). Most studies had very high statistical power (n = 13, 4.6%), while a smaller proportion had very low power (n = 6, 2.1%). Fewer studies fell into the high (n = 4, 1.4%), moderate (n = 2, 0.7%), or low (n = 3, 1.1%) categories.

We included 28 studies analyzing rs2032582 (c.2677G>T/A, p.Ala893Ser/Thr) in the final analysis. The studies were conducted between 2006 and 2021, and the majority were published in 2011. Most studies were conducted in China and India (7; 25.0% and 5; 17.9%, respectively). Half of the publications, 16 (57.0%), included patients with both focal and generalized epilepsy. Statistical power analysis revealed that 13 studies (46.4%) exhibited very high statistical power, and four (14.3%) demonstrated high power. Figure 6, Figure 7 and Figure 8 present the distribution of included studies by population, epilepsy type, and statistical power, respectively.

#### 3.2.2. Genotype Associations, Effect Sizes, and Required Sample Sizes for rs2032582

In very high-power studies, the rs2032582 TT genotype has been associated with ASM resistance in Tunisia [16], Malaysia [54], and Japan [17]. The AA and AT genotypes were more frequent in drug-resistant epilepsy patients in Pakistan [13]. Among the North Indian population, allele A was associated with a drug-resistant phenotype [55]. The G/T/A genotypes and haplotypes containing these genotypes were associated with drug resistance in the Han Chinese population [18]. Patients with the GT genotype had a significantly lower risk of developing pharmacoresistance in Croatia [22]. The GG genotype was associated with an ASM response in China [33]. The GG genotype was associated with a carbamazepine response, whereas the TT genotype was more likely to be resistant to carbamazepine in Malaysians [54]. In contrast, the GG genotype was associated with a drug-resistant phenotype in the Polish population [56]. In the high-power studies, the TT genotype was associated with antiseizure medication resistance in Jordanian females [38].

The effect sizes varied from 0.06, requiring a calculated sample size of over 2600 participants, to 0.92, with only 12 participants. The number of participants ranged from 83–580 and 86–460 in the very high and high statistical power studies, respectively. There were 100–391 participants in the low-power studies (Korea, India, Iran, Turkey, and Iraq). An increase in sample size would have required 27–85% to reach a statistical power of 0.8. The number of participants ranged from 34–388 in the very low-power studies (Taiwan, Spain, Brazil, China, and Egypt) and would have needed a 99–953% increase to achieve high statistical power. The genotype frequencies, effect sizes, and power analyses of the studies are presented in Table 2.

### 3.3. rs1128503 (c.1236C>T, p.Gly412=)

#### 3.3.1. Characteristics of Studies Included in the Analysis of rs1128503

**Figure 9:**
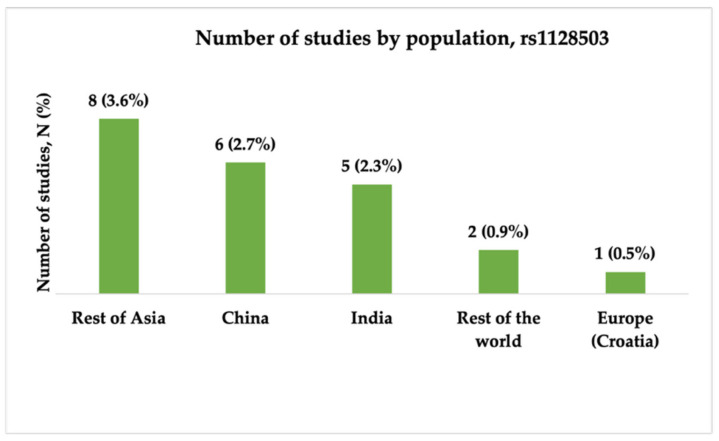
The number of included studies investigating the association between rs1128503 (c.1236C>T, p.Gly412=) and antiseizure medication resistance by population. “Rest of Asia” includes Malaysia, Vietnam, Korea, Taiwan, Iran, Pakistan, Jordan, and Japan; “Rest of the world” includes Brazil and Tunisia.

**Figure 10:**
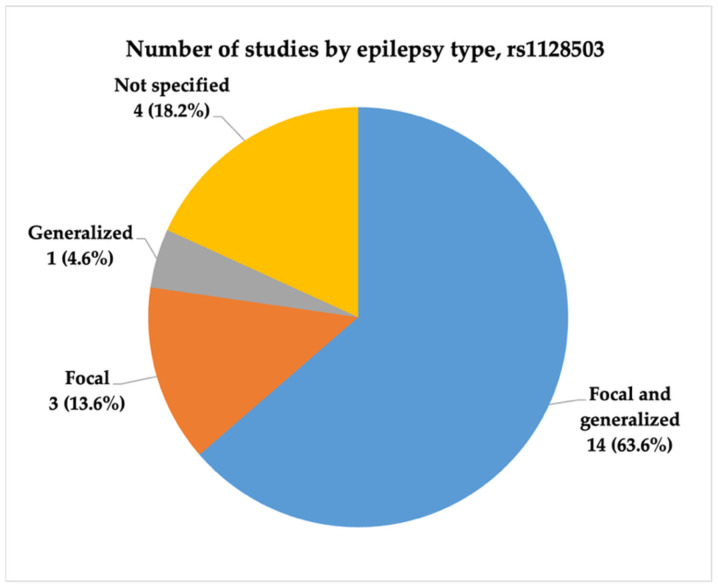
The distribution of included studies by epilepsy type for rs1128503 (c.1236C>T, p.Gly412=). Most studies (N = 14, 63.6%) investigated both focal and generalized epilepsy, while smaller proportions focused on focal (N = 3, 13.6%) or generalized (N = 1, 4.6%) epilepsy.

**Figure 11:**
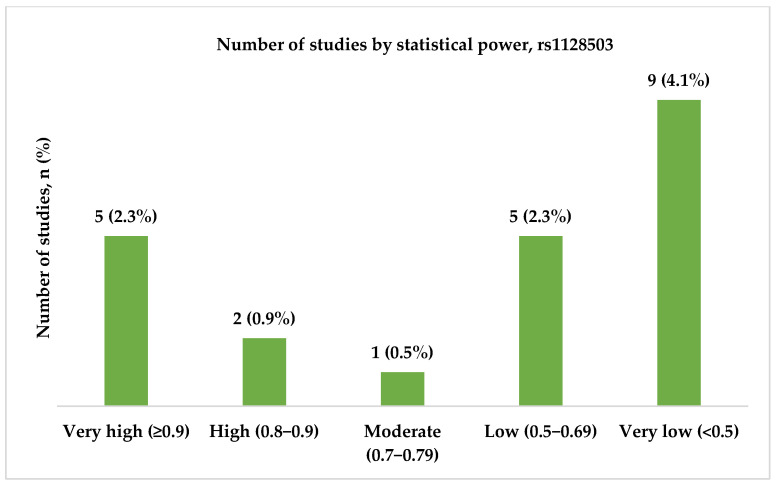
The distribution of included studies by statistical power for rs1128503 (c.1236C>T, p.Gly412=). Most studies had very low statistical power (N = 9, 4.1%), followed by very high (N = 5, 2.3%) and low (N = 5, 2.3%) power. Only a few studies showed high (N = 2, 0.9%) or moderate (N = 1, 0.5%) statistical power.

In the final analysis, we included 22 studies in relation to the rs1128503 (c.1236C>T, p.Gly412=) polymorphism. Several studies were conducted between 2006 and 2024, and most were published between 2009 and 2011. The majority of the studies were conducted in China (6, 27.3%) and India (5, 22.7%). Most of the studies (10, 47.6%) included both focal and generalized types of epilepsy, and six (28.6%) studies did not specify the epilepsy type. Only three (14.3%) studies had very high statistical power, and two (9.5%) had high statistical power. Figure 9, Figure 10 and Figure 11 present the distribution of the included studies by population, epilepsy type, and statistical power, respectively.

#### 3.3.2. Genotype Associations, Effect Sizes, and Required Sample Sizes for rs1128503

In the very high-power studies, the rs1128503 CC and CT genotypes were associated with drug resistance in female Iranian patients [58] and Pakistani patients [13], while the TT genotype was associated with drug resistance in Chinese patients [59]. There were no significant differences in the genotype frequencies of the rs1128503 SNP in Croatian epilepsy patients, except when analyzed within the G2677/C3435/C1236 haplotype. The GG/CC/CC genotype was significantly overrepresented among patients with drug resistance [22].

The effect sizes ranged from 0.04, requiring an estimated sample size of over 5700 participants, to as large as 0.64, detectable with just 24 participants. The number of participants included by the researchers varied from 99–332 and 100–227 in the very high-power and high-power studies. The low statistical group had 153–327 participants. An increase of 26–91% would have been needed to reach a study power of 0.8. In the very low-power group, the number of participants varied from 29–459, and the number of participants increased by 104–1681% to strengthen the study power. The genotype frequencies, effect sizes, and power analyses of the studies are presented in Table 3.

## 4. Discussion

There are 49 ABC genes in the human genome, arranged into seven subfamilies named A to G (ABCA ABCG) [60]. Most ABC genes encode membrane-bound proteins that transport molecules across cellular and intracellular membranes [61]. ABC transporters contain ATP-binding cassettes (given the acronym “ABC”) and utilize the energy released by ATP hydrolysis to drive diverse cellular processes through the transport of ions, sugars, amino acids, vitamins, peptides, polysaccharides, hormones, lipids, and xenobiotics [62]. ABCA2, ABCA3, ABCA6, ABCB1, ABCC1, ABCC3, ABCC10, ABCC12, and ABCG2 are associated with drug and multidrug resistance, whereas ABCB1 is the primary focus of drug-resistant epilepsy association studies [60].

### 4.1. ABCB1 Gene and P-Glycoprotein

**Figure 12:**
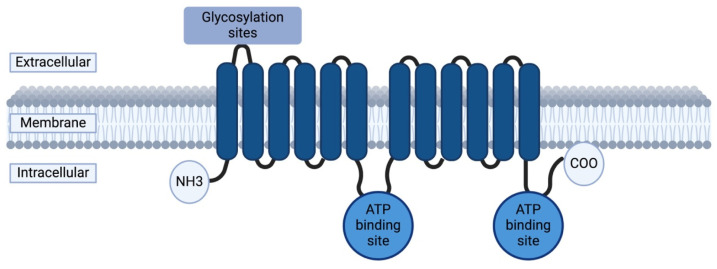
Schematic representation of the ATP-binding cassette subfamily B member 1 (ABCB1) transporter, also known as P-glycoprotein. Created in BioRender. Daškevičiūtė, A. (2025) https://BioRender.com/t07b794, accessed on 11 May 2025.

ABCB1 (MDR1, Multidrug Resistance Protein 1) is a subfamily B (MDR/TAP) member located on chromosome 7q21.12 [61]. The gene spans approximately 210 kb and consists of 29 exons, with the first two being part of the promoter region [62]. ABCB1 encodes a transmembrane protein named P-gp or multidrug resistance protein 1, which is composed of 1280 amino acids (approximately 170 kDa) [63]. P-gp has two halves comprising six hydrophobic transmembrane domains and one cytoplasmic ATP-binding domain [64]. The twelve transmembrane helices form a toroidal protein with an aqueous pore [65]. P-gp has two openings in the lipid bilayer that allow substrates to be extracted directly from the membrane upon passive diffusion into the cells (Figure 12) [66].

P-gp interacts with numerous substrates, exhibits high conformational flexibility [67], and uses ATP to actively transport substances out of the cell against their concentration gradients [68]. P-gp is expressed in different body tissues, including the gastrointestinal tract, liver, kidneys, testis, placenta, and brain [69]. P-gp is located on the apical surfaces of cells facing the lumen of enterocytes, hepatocytes, kidney tubule cells, and vascular lumens [70]. P-gp acts as a barrier and a protective mechanism against potentially toxic xenobiotics alongside xenobiotic-metabolizing enzymes [71].

The distribution and elimination of P-gp can directly influence the absorption, distribution, metabolism, excretion, and toxicity of drugs as it controls the rate of cellular uptake of foreign substances [66]. P-gp limits drug absorption due to its expression in the apical membrane of enterocytes, which promotes drug elimination into bile and urine as a result of its expression in the canalicular membrane of hepatocytes and the luminal membrane of tubule cells in the kidneys, respectively [71]. Finally, once the drug enters systemic circulation, the drug’s penetration into tissues such as the brain, testis, lymphocytes, and fetal circulation is limited by P-gp [71]. Known P-gp substrates include various antineoplastic agents, ASMs, β-adrenoceptor antagonists, calcium channel blockers, steroids, opioids, immunosuppressive drugs, HIV protease inhibitors, antiemetics, anthelmintics, antibiotics, lipid-lowering agents, and histamine H1 receptor antagonists, with 480 substrates identified so far [69].

P-gp was first described in Chinese hamster colchicine-resistant ovarian tissue cells in 1976 [63]. Thereafter, the role of pharmacoresistance in cancer cells has been studied extensively, and studies on P-gp-associated pharmacoresistance in other diseases have begun, including pharmacoresistant epilepsy [72]. In 1995, Tishler et al. were the first to report that the brain specimens of patients who are pharmacoresistant had >10 times P-gp mRNA levels than those with normal brains [73]. Immunohistochemistry for P-gp showed increased staining in the capillary endothelium and astrocytes, indicating that hyper-expression of P-gp leads to lower intraparenchymal ASM concentrations in the brain [73]. Sisodiya et al. hypothesized that P-gp’s hyper-expression in the aforementioned study may have been caused by seizures and exposure to ASM, which could have confounded the results. Therefore, Sisodiya et al. analyzed P-gp’s expression in ASM-naïve patients with malformations of cortical development and found that P-gp was overexpressed in glial cells and reactive astrocytes of the epileptogenic tissue of patients with dysembryoplastic neuroepithelial tumors, hippocampal sclerosis, and focal cortical dysplasia [74]. Sisodiya proposed that P-gp overexpression in astrocytes, which cover blood cells, may serve as a “second barrier” when the normal endothelial blood–brain barrier is disrupted during seizures [75]. Therefore, it remains unclear whether P-gp overexpression in the epileptogenic brain tissue of patients with pharmacoresistant epilepsy is the cause or consequence of drug-resistant epilepsy, uncontrolled seizures, chronic ASM treatment, or a combination of these factors [72]. A recent review indicated that P-gp and other ABC transporters induce pharmacokinetic changes in ASM by acting peripherally and induce pharmacodynamic changes by acting at a central level, mainly P-gp) [76].

### 4.2. ABCB1 Polymorphisms: Functional Impact on P-Glycoprotein

The most studied ABCB1 polymorphism is rs1045642 (c.3435C>T, p.Ile1145=) [74]. This silent substitution is translated into isoleucine, a hydrophobic residue at position 2677 (motif: AUG in AUU) [74]. Kimchi-Sarfaty et al. demonstrated that rs1045642 changed the timing of co-translational folding and the insertion of P-gp into the cell membrane, ultimately leading to altered substrate specificity despite not altering the amino acid sequence of the encoded P-gp [77]. This suggests that c.3435C>T causes functional alterations in the protein, potentially by affecting rhythm translation [64].

rs2032582 (c.2677G>T/A, p.Ala893Ser/Thr) is a triallelic SNP located in exon 21 and is situated on the intracellular side of P-gp after the transmembrane region 10 [74]. rs2032582 contains a nonsynonymous amino acid change from alanine (Ala) at codon 893 to serine/threonine (Ser/Thr) [74]. Furthermore, it could affect the co-translation of amino acids before the intracellular loop-forming domains TM9 and TM10, thereby affecting the drug-induced ATPase activity of P-gp [64].

rs1128503 (c.1236C>T, p.Gly412=) is located in exon 12 and may affect the co-translational folding of TM6, a domain within the P-gp drug-binding pocket that is essential for UIC2 antibody recognition and substrate binding [78]. rs1128503 can alter protein expression, function, and mRNA stability [64]. The effect of the 1236C>T mutation could have resulted from the use of a rarer codon (from GGC to GGT) [64].

Linkage analysis confirmed that the most frequent haplotype was the 3435C>T polymorphism combined with 2677G>T/A and/or 1236C>T [64]. Despite the impact of the independence of the SNPs, using the haplotype (1236C>T, 2677G>T, 3435C>T) may enable the detection of a linkage to a phenotype rather than studying only the 3435C>T polymorphism alone. These SNPs may produce a more salient phenotype when they are present together [64]. In a study by Hung et al., haplotype analysis demonstrated that patients with CGC, TGC, and TTT haplotypes, as well as those with the haplotypic combinations CGC/CGC, CGC/TGC, CGC/TTT, and TGC/TTT, were more likely to be drug-resistant [79]. Zimprich et al. detected a strong linkage disequilibrium between these three SNPs. They discovered that homozygous carriers of the CGC haplotype were significantly more common in groups with higher pharmacoresistance. In contrast, weak associations were found with separate analyses of the three SNPs [80]. Kwan et al. found that ABCB1 intronic polymorphism rs3789243, coding polymorphism 2677, and haplotypes containing them may be associated with drug resistance [18]. Similarly, Sporiš et al. found that the haplotype G2677/C3435/C1236 was significantly overrepresented in patients who are drug-resistant [22].

### 4.3. Study Design and Challenges in ABCB1 Pharmacogenetic Research

Several studies have been conducted to establish the role of ABCB1 polymorphisms in drug-resistant epilepsy, with inconsistent results. In genetic studies, significance testing is the most frequently used method to evaluate statistical hypotheses. However, *p*-values are difficult to interpret without considering their statistical power, and an insignificant test can result from both the absence of effect and insufficient statistical power [81]. We evaluated existing genetic association studies in depth, focusing on the effect sizes, power analysis, and the required sample sizes for each study. Based on our analysis, high and very high statistical power for the rs1045642 (c.3435C>T, p.Ile1145=) and rs2032582 (c.2677G>T/A, p.Ala893Ser/Thr) polymorphisms was achieved in 58.0 and 60.7%, respectively, whereas rs1128503 achieved 31.8% in the high-power studies. rs1045642 showed the strongest statistical power overall, with many high-quality analyses. In contrast, rs2032582 and especially rs1128503 were predominantly investigated in underpowered studies, highlighting the need for larger, more robust research to validate their associations. Low-powered studies can act as pilot or exploratory studies; however, they experience challenges in interpreting the results due to the risk of false negatives, false positives, and overestimated effects.

Moreover, most studies on SNP- and ASM-resistance analyses included mixed-patient samples comprising both focal and generalized epilepsy. This was true in 82.0, 57.0, and 64.0% of the studies for rs1045642, rs2032582, and rs1128503, respectively. These studies presented results for focal and generalized epilepsy without splitting them into two groups. Therefore, this raises the question of whether the two distinct epilepsy types can be analyzed together in the same sample group, as generalized epilepsy has a complex genetic background. In our opinion, analyzing these epilepsy types in at least two distinct groups is preferable.

**Figure 13:**
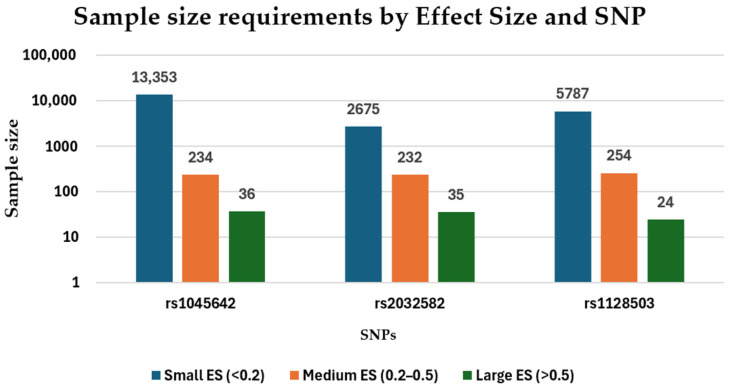
Visual representation of the required sample sizes for various effect sizes (ESs) in studies analyzing the relationship between rs1045642 (c.3435C>T, p.Ile1145=), rs2032582 (c.2677G>T/A, p.Ala893Ser/Thr), and rs1128503 (c.1236C>T, p.Gly412=) and antiseizure medication response.

The effect sizes (ESs) observed in this analysis varied widely, highlighting the importance of significantly larger sample sizes to achieve statistical significance for small effect sizes, whereas larger effect sizes can be detected with smaller sample sizes. Regarding the rs1045642 SNP, small ESs (ES < 0.2) required sample sizes from 234 to 13,353 participants to achieve a study power of ≥0.8. Medium ESs (ES = 0.2–0.5) required sample sizes between 40 and 234 participants, and large effect sizes (ES > 0.5) required between 9 and 36 participants. Regarding rs2032582 SNP, small ESs required sample sizes ranging from 357 to 2675 participants, medium ESs required 41 to 232 participants, and large ESs required 12 to 35 participants. Regarding rs1128503, small effect sizes required a large number of participants, ranging from 267 to 5.787, medium ESs required 63 to 254, and one study with a large ES required 24 participants (Figure 13).

**Figure 14:**
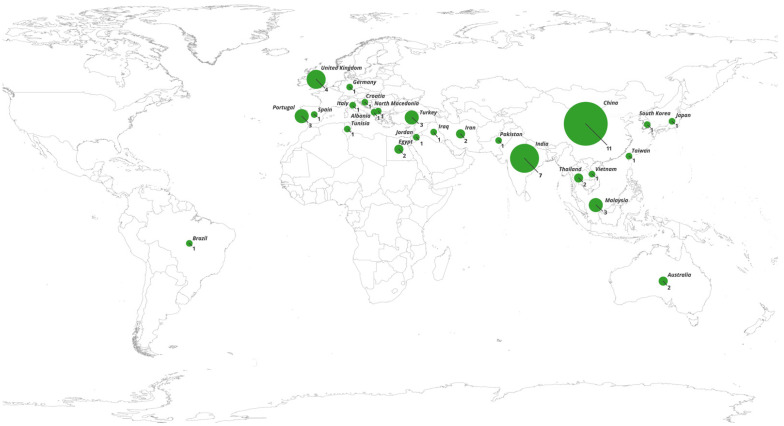
Visual representation of the countries that evaluated ABCB1 SNPs (rs1045642, rs2032582, and rs1128503) and their association with antiseizure medication responses. Circle size and number indicate the number of included studies per country. Map created using the Free and Open Source QGIS (QGIS Development Team, https://qgis.org, accessed on 12 May 2025).

Our review revealed a shortage of studies on European, African, and Middle Eastern populations; South Asian populations (other than India); Southeast Asian countries beyond Malaysia and Thailand; and Latin American countries (Figure 14). rs1045642 was the most studied polymorphism, with consistent publications ranging from 2003–2024. The years 2009 and 2011 had the most studies on the relationship between single-nucleotide polymorphisms and antiseizure medication response; however, there was a noticeable decline in 2015, which was probably due to attention being driven to other emerging fields in epilepsy research.

### 4.4. Genotype Distribution of ABCB1 Polymorphisms Across Populations

**Figure 15:**
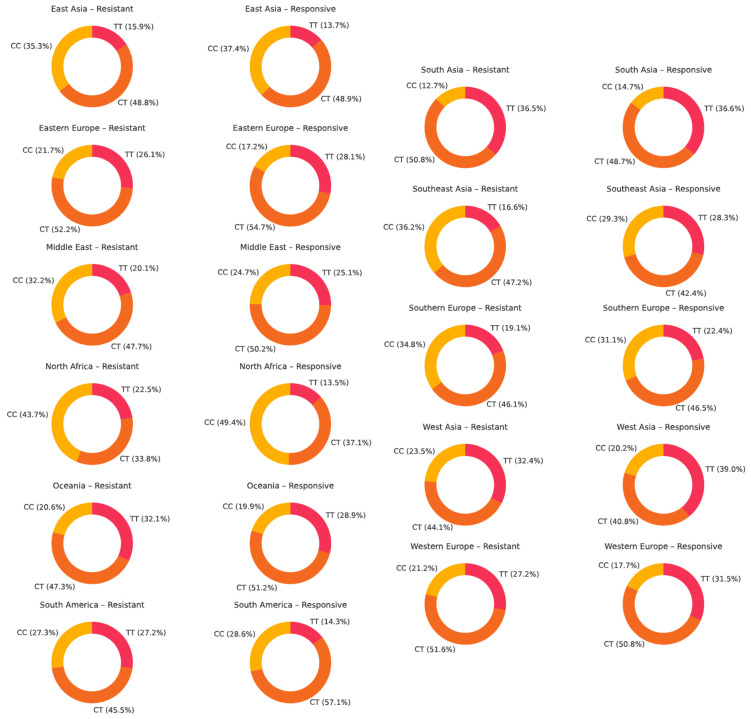
The genotype distribution of ABCB1 rs1045642 (c.3435C>T, p.Ile1145=) across geographic regions in patients with drug-resistant and drug-responsive epilepsy.

The genotype frequencies of rs1045642 (c.3435C>T, p.Ile1145=) across continental populations in antiseizure medication-resistant versus responsive epilepsy groups are represented in Figure 15. The heterozygous CT genotype is the most frequent, accounting for 40–55% of individuals, with slightly higher frequencies observed in drug-responsive populations. Notable variability is observed between populations: for example, the TT genotype is more frequent in Oceania and West Asia among drug-resistant patients (32.0% and 32.4%, respectively), while in Western Europe, the TT genotype is more prevalent among responsive individuals (31.5% vs. 27.2%). Conversely, Eastern Europe shows a lower TT frequency in both groups.

**Figure 16:**
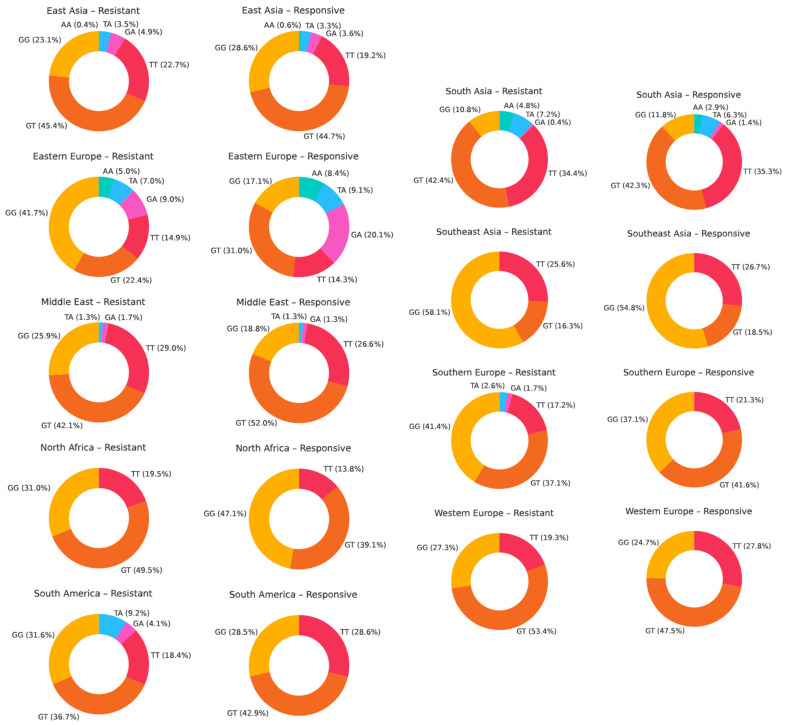
The genotype distribution of ABCB1 rs2032582 (c.2677G>T/A, p.Ala893Ser/Thr) across geographic regions in patients with drug-resistant and drug-responsive epilepsy.

For rs2032582 (c.2677G>T/A, p.Ala893Ser/Thr), across nearly all populations, the heterozygous GT genotype is the most prevalent in both drug-resistant and drug-responsive groups. However, its dominance is slightly more consistent in the drug-resistant groups, where GT often comprises 45–50% of individuals, such as in East Asia, South Asia, and North Africa. The GT genotype frequency is still high in drug-responsive groups, but slightly more variable across regions. Conversely, the wild-type GG genotype is more commonly observed in drug-responsive individuals in regions like North Africa, Southeast Asia, and South America. Notably, certain minor genotypes (e.g., TA, GA, and AA) appear at low frequencies, reflecting ethnic diversity (Figure 16).

**Figure 17:**
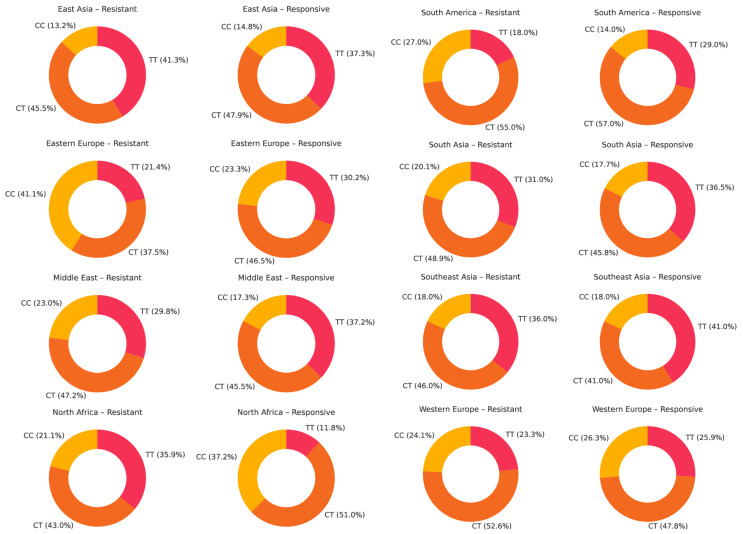
The genotype distribution of ABCB1 rs1128503 (c.1236C>T, p.Gly412=) across geographic regions in patients with drug-resistant and drug-responsive epilepsy.

For rs1128503 (c.1236C>T, p.Gly412=), the heterozygous CT genotype is the most prevalent, especially among drug-responsive individuals, where it typically accounts for 45–55% of cases, most notably in regions such as North Africa, South America, and Western Europe. In drug-resistant populations, the CT genotype also dominates but displays slightly more variation across regions. The TT genotype is more prevalent in drug-resistant individuals in areas like East Asia and the Middle East, suggesting a possible association with resistance. Conversely, the CC genotype tends to be more frequent in drug-responsive groups, particularly in North Africa and Southeast Asia (Figure 17).

### 4.5. ABCB1 Polymorphisms and Antiseizure Medication Resistance

**Figure 18:**
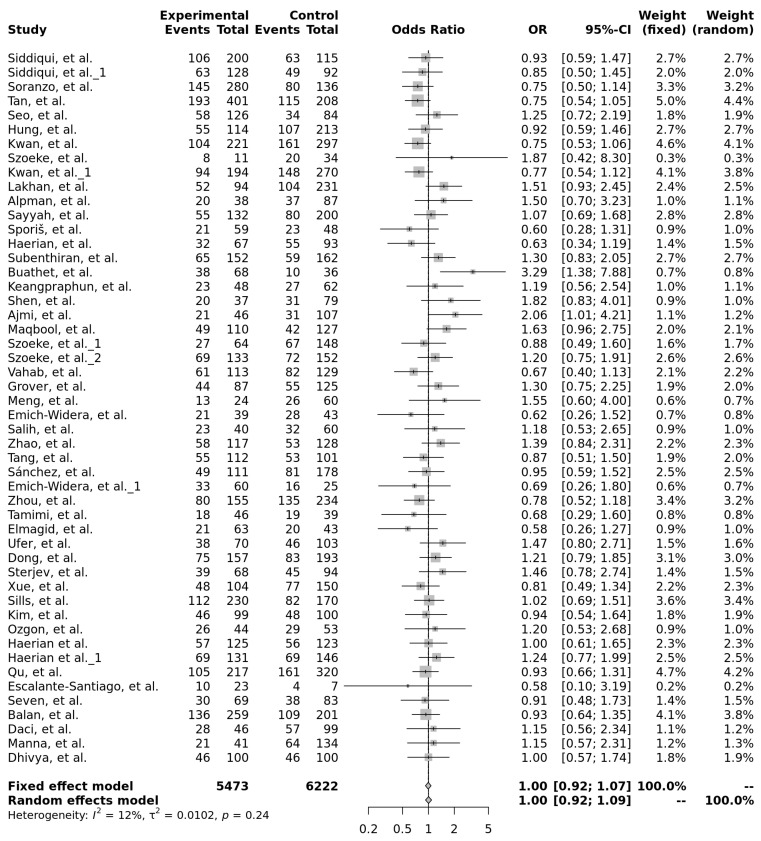
Forest plot of the association between the TT and CC genotypes of rs1045642 (c.3435C>T, p.Ile1145=) and drug-resistant epilepsy, using both fixed-effect and random-effects models. The last row shows the total number of participants in the experimental and control groups, the pooled odds ratio with 95% confidence interval, and the overall study weight. Studies included: [9,10,11,12,13,14,15,16,17,18,19,20,21,22,23,24,26,27,28,29,30,31,32,33,34,35,36,37,38,39,40,41,42,43,44,45,46,47,48,49,50,51,52,53].

For rs1045642 (c.3435C>T, p.Ile1145=), 14 out of 29 (48%) studies with very high and high power showed a significant association with antiseizure medication resistance, with seven (24%) of them having the CC genotype (Figure 18). Although not statistically significant, in forest plots, the TT genotype showed a trend toward a protective effect compared to the CC genotype (OR = 0.92, 95% CI: 0.75–1.13), suggesting it may be associated with lower odds of drug-resistant epilepsy (Figure 15). Forest plots were generated for multiple genetic models—including allele contrast (T vs. C), recessive (TT vs. TC + CC), dominant (TT + TC vs. CC), and over-dominant (TC vs. TT + CC)—as well as direct genotype comparisons (TT vs. CC, TT vs. TC, and TC vs. CC), none of which reached statistical significance (Table 4). The heterogeneity of the studies ranged from low to moderate (I^2^ = 12–64%).

**Figure 19:**
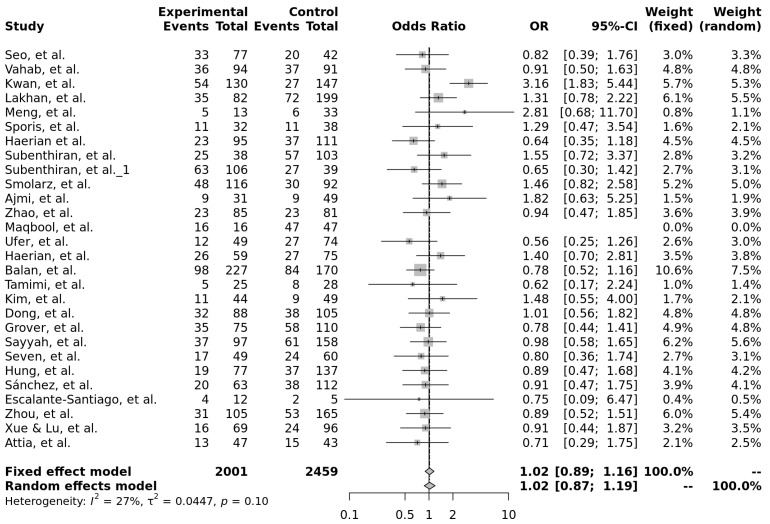
Forest plot of the association between the TT and TC genotypes of rs2032582 (c.2677G>T/A, p.Ala893Ser/Thr) and drug-resistant epilepsy, using both fixed-effect and random-effects models. The last row shows the total number of participants in the experimental and control groups, the pooled odds ratio with 95% confidence interval, and the overall study weight. Studies included: [11,12,13,15,16,17,19,22,27,28,29,30,31,33,35,37,38,40,41,43,45,48,49,50,54,56,57].

For rs2032582 (c.2677G>T/A, p.Ala893Ser/Thr), 9 out of 17 (53%) of the very high and high statistical power studies found a significant association of this SNP and drug-resistant epilepsy. Most of the studies (4; 24%) found the TT genotype associated with antiseizure medication resistance. In the forest plot analysis, none of the tested genetic models reached statistical significance; however, the comparison between TT and TC genotypes showed a potential trend toward an association with drug-resistant epilepsy. In this model, individuals with the TT genotype had slightly increased odds of resistance compared to the TC carriers (OR = 1.02, 95% CI: 0.87–1.19), with low heterogeneity across the studies (I^2^ = 27%, *p* = 0.10), Figure 19. A summary of the genetic model meta-analyses is presented in Table 5.

**Figure 20:**
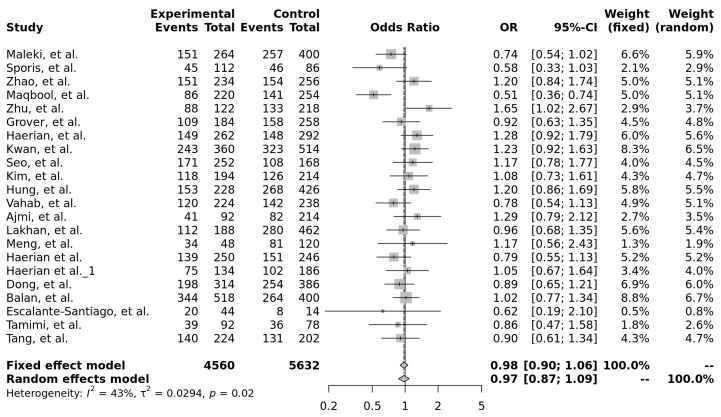
Forest plot of the association between the T and C alleles of rs1128503 (c.1236C>T, p.Gly412=) and drug-resistant epilepsy, using both fixed-effect and random-effects models. The last row shows the total number of participants in the experimental and control groups, the pooled odds ratio with 95% confidence interval, and the overall study weight. Studies included: [12,13,16,17,19,22,27,28,29,30,31,33,34,38,41,45,48,50,58,59].

For rs1128503 (c.1236C>T, p.Gly412=), three out of seven (43%) high and very high-power studies showed a significant association with drug-resistant epilepsy, with two (29%) of the studies having CC and CT genotypes. Although not statistically significant, the allele contrast model (T vs. C) showed a trend toward a protective effect of the T allele against drug-resistant epilepsy (OR = 0.97, 95% CI: 0.87–1.09). This suggests that the T allele may be associated with a lower risk of antiseizure medication resistance compared to the C allele, warranting further investigation (Figure 20). In the forest plot analysis, none of the tested genetic models reached statistical significance (Table 6).

## 5. Conclusions

1. High and very high statistical power for rs1045642 (c.3435C>T, p.Ile1145=), rs2032582 (c.2677G>T/A, p.Ala893Ser/Thr), and rs1128503 (c.1236C>T, p.Gly412=) polymorphisms was achieved only in 58.0, 60.7, and 31.8% of the studies, respectively. Considering both the effect sizes and statistical power when designing genetic association studies is essential, as they influence the reliability and interpretability of the results.
2. The effect sizes (ES) of rs1045642, rs2032582, and rs1128503 ranged from 0.03–1.04, 0.06–0.92, and 0.04–0.64, respectively. The required sample sizes for rs1045642, rs2032582, and rs1128503 ranged from 9–13,000, 12–2600, and 24–5700 participants, respectively.
3. There is a shortage of studies on European, African, and Middle Eastern populations, and South Asian, Southeast Asian, and Latin American countries. Expanding research into a wider range of populations could help identify population-specific genetic markers, improve global understanding, and guide treatment strategies for epilepsy.
4. The meta-analyses did not identify statistically significant associations between any of the three ABCB1 polymorphisms (rs1045642, rs2032582, or rs1128503) and antiseizure medication resistance.
5. For rs1045642, a non-significant trend toward a protective effect of the TT genotype was observed. The CT genotype was most prevalent, especially among drug-responsive individuals. For rs2032582, the TT genotype showed a weak tendency toward resistance, but no consistent association was found. GT was the most common genotype, while GG was more frequent in responsive patients in some regions. For rs1128503, no such genotype associations were detected. The TT genotype was slightly more common in drug-resistant groups, and CT remained the most prevalent, particularly in responsive populations.
6. Future studies investigating the association between ABCB1 polymorphisms and antiseizure medication resistance should consider stratifying drug-resistant epilepsy patients by epilepsy type.
7. Since 2015, there has been a decline in studies on the single-nucleotide polymorphisms rs1045642, rs2032582, and rs1128503 in relation to antiseizure medication resistance. Despite the reduced research attention to these SNPs, they still have considerable clinical potential, particularly if investigated through robust, large-scale studies and haplotype analyses, which could be crucial for clarifying their role in ASM resistance.

## Figures and Tables

**Table 1:** The rs1045642 (c.3435C>T, p.Ile1145=) genotype frequencies, effect sizes, study power, and required sample size to achieve power (1 − β) = 0.8.

Author	Year	Population	Epilepsy Type	Drug Resistant, N	CC, N (%)	CT, N (%)	TT, N (%)	Drug Responsive, N	CC, N (%)	CT, N (%)	TT, N (%)	Results	G-Power Calculations		
													Effect Size	Power 1 − β	Needed Sample Size
Very high statistical power															
Siddiqui, et al. [9]	2003	UK	Focal and generalized	200	55 (27.5%)	106 (53%)	39 (19.5%)	115	18 (15.7%)	63 (54.8%)	34 (29.6%)	DRE patients were more likely to have the CC genotype than the TT genotype (*p* = 0.006).	0.32	1.000	93
Siddiqui et al. [9]	2003	India	Focal and generalized	128	11 (8.6%)	63 (50%)	54 (42%)	92	21(23.0%)	49 (53.0%)	22 (24%)	No significant association with ASM resistance.	0.56	1.000	31
Soranzo et al. [21]	2004	UK	Not specified	280 * (* 286)	73 (25.5%)	145 (50.7%)	62 (21.7%)	136 * (135)	20 (14.8%)	80 (59.3%)	36 (26.7%)	The C allele was significantly overrepresented in the DRE group (*p* = 0.032).	0.26	0.999	143
Tan et al. [25]	2004	Australia	Focal and generalized	401	75 (18.7%)	193 (48.1%)	133 (33.2%)	208	37 (17.8%)	115 (55.3%)	56 (26.9%)	No significant association with ASM resistance.	0.15	0.930	416
Seo et al. [17]	2006	Japan	Focal and generalized	126	34 (27.0%)	58 (46.0%)	34 (27.0%)	84	36 (42.9%)	34 (40.5%)	14 (16.7%)	DRE had higher frequencies of the TT genotype (*p* = 0.027).	0.37	0.999	70
Hung et al. [12]	2007	Taiwan	Focal and generalized	114	40 (35%)	55 (48.0%)	19 (17.0%)	213	39 (18.0%)	107 (50.0%)	67 (32.0%)	DRE patients were more likely to have the CC genotype (*p* < 0.001).	0.46	1.000	46
Kwan et al. [19]	2007	China	Focal and generalized	221	80 (36.2%)	104 (47.1%)	37 (16.7%)	297	114 (38.4%)	161 (54.2%) *	22 (7.4%)	DRE patients were more likely to have the TT genotype (*p* = 0.0009).	0.25	1.000	151
Szoeke et al. [26]	2009	Hong Kong	Focal and generalized	11	1 (9.1%)	8 (72.7%)	2 (18.2%)	34	13 (38.2%)	20 (58.8%)	1 (2.9%)	No significant association with ASM resistance.	1.04	1.000	9
Kwan et al. [18]	2009	Han Chinese	Focal and generalized	194	71 (36.6%)	94 (48.5%)	29 (14.9%)	270	101 (37.4%)	148 (54.8%)	21 (7.8%)	DRE patients were more likely to have the TT genotype (*p* = 0.04).	0.21	0.983	229
Lakhan et al. [27]	2009	North India	Focal and generalized	94	9 (9.6%)	52 (55.3%)	33 (35.1%)	231	38 (16.5%)	104 (45.0%)	89 (38.5%)	No significant association with ASM resistance.	0.27	0.994	134
Alpman et al. [23]	2010	Turkey	Focal and generalized	38 * (39)	6 (15.4%)	20 (51.3%)	12 (30.8%)	87 * (92)	26 (28.3%)	37 (40.2%)	24 (26.1%)	No significant association with ASM resistance.	0.39	0.979	65
Sayyah et al. [11]	2011	Iran	Focal and generalized	132	34 (25.7%)	55 (41.6%)	43 (32.6%)	200	32 (16.0%)	80 (40.0%)	88 (44.0%)	DRE patients had a higher frequency of the CC genotype (*p* = 0.01).	0.28	0.997	125
Sporiš et al. [22]	2011	Croatia	Focal and generalized	59	19 (32.2%)	21 (35.6%)	19 (32.2%)	48	7 (14.6%)	23 (47.9%)	18 (37.5%)	No significant association with ASM resistance.	0.38	0.954	66
Haerian et al. [28]	2011	Indian	Focal and generalized	67	23 (34%)	32 (48%)	12 (18%)	93	17 (18%)	55 (59%)	21 (23%)	No significant association with ASM resistance.	0.39	0.976	85
Subenthiran et al. [15]	2013	Malaysia	Focal	152 * (162)	51 (34%)	65 (42.8%)	36 (23.7%)	162	35 (21.6%)	59 (36.4%)	68 (42.0%)	TT genotype was associated with ASM resistance (*p* = 0.007).	0.40	1.000	62
Buathet et al. [20]	2013	Thailand	Focal and generalized	68	19 (27.9%)	38 (55.9%)	11 (16.2%)	36	11 (30.6%)	10 (27.8%)	15 (41.7%)	CT and CC genotypes were associated with ASM resistance (*p* = 0.001 and *p* = 0.036, respectively).	0.74	1.000	18
Keangpraphun et al. [10]	2015	Thailand	Focal and generalized	48	19 (39.6%)	23 (47.9%)	6 (12.5%)	62	19 (30.6%)	27 (43.5%)	16 (25.8%)	DRE patients had a higher frequency of the CC genotype (*p* = 0.039).	0.41	0.976	59
Shen et al. [14]	2017	Han Chinese	Focal and generalized	37	9 (24.3%)	20 (54.1%)	8 (21.6%)	79	39 (49.4%)	31 (39.2%)	9 (11.4%)	Patients with the CC genotype were more likely to be ASM-responsive than those with CT (*p* = 0.025) and TT (*p* = 0.022) genotypes.	0.59	1.000	28
Ajmi et al. [16]	2018	Tunisia	Focal and generalized	46	19 (41.3%)	21 (45.7%)	6 (13%)	107	70 (65.4%)	31 (29.0%)	6 (5.6%)	TT genotype was more frequent in DRE patients (*p* = 0.017).	0.49	1.000	40
Maqbool et al. [13]	2021	Pakistan	Idiopathic generalized epilepsy	110	39 (35.5%)	49 (44.5%)	22 (20.0%)	127	33 (26.0%)	42 (33.1%)	52 (40.9%)	CC genotype was strongly associated with DRE (*p* = 0.0009).	0.52	1.000	36
High statistical power															
Szoeke et al. [26]	2009	Australia	Focal and generalized	64	21 (32.8%)	27 (42.2%)	16 (25.0%)	148	34 (23.0%)	67 (45.3%)	47 (31.7%)	No significant association with ASM resistance.	0.22	0.838	193
Szoeke et al. [26]	2009	Scotland	Focal and generalized	133	20 (15%)	69 (51.9%)	44 (33.1%)	152	34 (22.4%)	72 (47.4%)	46 (30.3%)	No significant association with ASM resistance.	0.21	0.887	227
Vahab et al. [29]	2009	India	Focal and generalized	113	3 (2.65%)	61 (53.98%)	49 (43.36%)	129	4 (3.1%)	82 (63.57%)	43 (33.33%)	No significant association with ASM resistance.	0.20	0.815	234
Grover et al. [30]	2010	India	Focal and generalized	87 * (95)	13 (14.9%)	44 (50.6%)	30 (34.5%)	125 * (133)	14 (11.2%)	55 (44.0%)	56 (44.8%)	No significant association with ASM resistance.	0.22	0.827	199
Meng et al. [31]	2011	China	Focal and generalized	24	6 (25%)	13 (54.2%)	5 (20.8%)	60	24 (40.0%)	26 (43.3%)	10 (16.7%)	No significant association with ASM resistance.	0.35	0.819	81
Emich-Widera et al. [32]	2014	Poland	Focal	39	8 (20.5%)	21 (53.8%)	10 (25.6%)	43	2 (4.7%)	28 (65.1%)	13 (30.2%)	No significant association with ASM resistance.	0.39	0.898	63
Salih et al. [24]	2020	Iraq	Focal and generalized	40	13 (32.5%)	23 (57.5%)	4 (10%)	60	11 (18.33%)	32 (55.33%)	17 (28.33%)	DRE patients had a higher frequency of CT + CC genotypes (*p* = 0.019).	0.27	0.802	130
Zhao et al. [33]	2020	China	Focal and generalized	117	32 (27%)	58 (50%)	27 (23%)	128	46 (36%)	53 (41%)	29 (23%)	No significant association with ASM resistance.	0.215	0.863	209
Tang et al. [34]	2024	Vietnam	Focal and generalized	112	47 (42.0%)	55 (49.1%)	10 (8.9%)	101	33 (32.7%)	53 (52.5%)	15 (14.9%)	No significant association with ASM resistance.	0.26	0.802	141
Moderate statistical power															
Sánchez et al. [35]	2010	Spain	Focal and generalized	111	40 (36%)	49 (44.1%)	22 (18.8%)	178	52 (29.2%)	81 (45.5%)	45 (25.3%)	In DRE patients, the CC genotype was significantly more frequent than the TT genotype (*p* = 0.019).	0.17	0.732	338
Emich-Widera et al. [36]	2013	Poland	Focal	60	9 (15.0%)	33 (55.0%)	18 (30.0%)	25	1 (4.0%)	16 (64.0%)	8 (32.0%)	No significant association with ASM resistance.	0.31	0.730	100
Zhou et al. [37]	2015	China	Focal and generalized	155 * (156)	55 (35.5%)	80 (51.6%)	20 (12.9%)	234 * (235)	79 (33.8%)	135 (57.7%)	20 (8.5%)	No significant association with ASM resistance.	0.15	0.769	419
Tamimi et al. [38]	2021	Jordan	Not specified	46	21 (45.7%)	18 (39.1%)	7 (15.2%)	39	12 (30.8%)	19 (48.7%)	8 (20.5%)	DRE was 14 times more likely in females with the CC genotype compared to those with the TT genotype (*p* = 0.028).	0.30	0.700	107
Elmagid et al. [39]	2021	Egypt	Focal and generalized	63	32 (50.8%)	21 (33.3%)	10 (15.9%)	43	18 (41.9%)	20 (46.5%)	5 (11.6%)	No significant association with ASM resistance.	0.28	0.742	122
Low statistical power															
Ufer et al. [40]	2009	Germany	Focal and generalized	70	10 (14.3%)	38 (54.3%)	22 (31.4%)	103	20 (19.4%)	46 (44.7%)	37 (35.9%)	No significant association with ASM resistance.	0.20	0.672	230
Dong et al. [41]	2011	China	Focal and generalized	157	64 (40.7%)	75 (47.8%)	18 (11.5%)	193	82 (42.5%)	83 (43.0%)	28 (14.5%)	No significant association with ASM resistance.	0.12	0.481	712
Sterjev et al. [42]	2012	Macedonia	Focal and generalized	68	15 (22.0%)	39 (57.0%)	14 (21.0%)	94	25 (26.6%)	45 (47.9%)	24 (25.5%)	No significant association with ASM resistance.	0.19	0.579	264
Xue et al. [43]	2016	Han Chinese	Focal and generalized	104	43 (41.35%)	48 (46.15%)	13 (12.5%)	150	61 (40.67%)	77 (51.33%)	12 (8.00%)	No significant association with ASM resistance.	0.15	0.553	437
Very low statistical power															
Sills et al. [44]	2005	Scotland	Focal and generalized	230	41 (17.8%)	112 (48.7%)	77 (33.5%)	170	32 (18.8%)	82 (48.2%)	56 (32.9%)	No significant association with ASM resistance.	0.03	0.072	13353
Kim et al. [45]	2006	Korea	Not specified	99	47 (47.5%)	46 (46.5%)	6 (6.1%)	100	45 (45.0%)	48 (48.0%)	7 (7.0%)	No significant association with ASM resistance.	0.06	0.106	2780
Ozgon et al. [46]	2008	Turkey	Focal and generalized	44	13 (29.6%)	26 (59.1%)	5 (11.3%)	53	16 (30.3%)	29 (54.7%)	8 (15.0%)	No significant association with ASM resistance.	0.12	0.178	624
Haerian et al. [28]	2011	Malays	Focal and generalized	125	47 (38%)	57 (46%)	21 (17%)	123	44 (36%)	56 (45%)	23 (19%)	No significant association with ASM resistance.	0.06	0.119	2830
Haerian et al. [28]	2011	Chinese	Focal and generalized	131	39 (30%)	69 (53%)	23 (18%)	146	49 (34%)	69 (47%)	28 (19%)	No significant association with ASM resistance.	0.10	0.318	901
Qu et al. [47]	2012	China	Focal and generalized	217	81 (37.3%)	105 (48.4%)	31 (14.3%)	320	116 (36.3%)	161(50.3%)	43 (13.4%)	No significant association with ASM resistance.	0.04	0.119	6097
Escalante et al. [48]	2014	Brazil	Focal	22	30.0%	45.0%	25.5%	7	28.6%	57.1%	14.3%	No significant association with ASM resistance.	0.28	0.253	122
Seven et al. [49]	2014	Turkey	Focal and generalized	69	17 (25.0%)	30 (43.5%)	22 (32.0%)	83	22 (26.5%)	38 (46.0%)	23 (28.0%)	No significant association with ASM resistance.	0.09	0.155	1174
Balan et al. [50]	2014	India	Focal and generalized	259	12 (0.05)	136 (52.5%)	111 (0.43)	201	12 (6.0%)	109 (54.0%)	80 (40.0%)	No significant association with ASM resistance.	0.08	0.338	1399
Daci et al. [51]	2015	Albania	Focal and generalized	46	8 (17.4%)	28 (60.9%)	10 (21.7%)	99	18 (18.2%)	57 (57.6%)	24 (24.2%)	No significant association with ASM resistance.	0.07	0.109	1914
Manna et al. [52]	2015	Italy	Focal	41	13 (31.7%)	21 (51.2%)	7 (17.1%)	134	45 (33.6%)	64 (47.8%)	25 (18.7%)	No significant association with ASM resistance.	0.07	0.122	1917
Dhivya et al. [53]	2024	South India	Focal and generalized	100	16 (16%)	46 (46%)	38 (38%)	100	19 (19%)	46 (46%)	35 (35%)	No significant association with ASM resistance.	0.09	0.188	1206

* The first number represents the calculated sum of the genotypes, whereas the numbers in parentheses correspond to the values provided in the reference article.

**Table 2:** The rs2032582 (c.2677G>T/A, p.Ala893Ser/Thr) genotype frequencies, effect sizes, study power, and required sample size to achieve power (1 − β) = 0.8.

Author	Year	Population	Epilepsy Type	Drug Responsiveness	Number of Participants, N	GG, N (%)	GT, N (%)	TT, N (%)	GA, N (%)	TA, N (%)	AA, N (%)	Conclusion	G-Power Calculations		
													Effect Size	Power 1 − β	Needed Sample Size
Very high statistical power															
Seo et al. [17]	2006	Japan	Focal and generalized	Drug resistant, N	126	18 (14.3%)	44 (34.9%)	33 (26.2%)	15 (11.9%)	15 (11.9%)	1 (0.8%)	DRE patients were more likely to have the TT genotypes than the GG genotypes (*p* = 0.049).	0.40	0.998	81
				Drug responsive, N	84	22 (26.2%)	22 (26.2%)	20 (23.8%)	8 (9.5%)	10 (11.9%)	2 (2.4%)				
Vahab et al. [29]	2009	India	Unspecified	Drug resistant, N	110 * (* 112)	16 (14.54%)	58 (52.73%)	36 (32.73%)	NR	NR	NR	No significant association with ASM resistance.	0.26	0.948	145
				Drug responsive, N	119	28 (23.53%)	54 (45.38%)	37 (31.09%)	NR	NR	NR				
Kwan et al. [18]	2009	Han Chinese	Unspecified	Drug resistant, N	174 * (194)	44 (25.3%)	76 (43.7%)	54 (31.0%)	NR	NR	NR	T/A genotypes were significantly associated with ASM resistance (*p* = 0.020).	0.49	1.000	41
				Drug responsive, N	251 * (270)	104 (41.4%)	120 (47.8%)	27 (10.8%)	NR	NR	NR				
Lakhan et al. [27]	2009	North India	Focal and generalized	Drug resistant, N	97	10 (10.8%) *	47 (50.0%)	35 (37.2%)	0 (0%)	2 (2.1%)	NR	No significant association with ASM resistance.	0.29	0.994	148
				Drug responsive, N	234	16 (6.9%)	127 (55.0%)	72 (31.2%)	2 (0.9%)	14 (6.1%)	NR				
Meng et al. [31]	2011	Chinese Han	Focal and generalized	Drug resistant, N	24	5 (20.8%)	8 (33.3%)	5 (20.8%)	0 (0.00%)	6 (25%)	0 (0.00%)	No significant association with ASM resistance.	0.55	0.989	44
				Drug responsive, N	59	12 (20.3%)	27 (45.8%)	6 (10.2%)	3 (5.1%)	8 (13.6%)	3 (5.1%)				
Sporis et al. [22]	2011	Croatia	Focal	Drug resistant, N	58	26 (44.8%)	21 (36.2%)	11 (19%)	NR	NR	NR	Patients with the GT genotype had a statistically significantly lower chance for ASM resistance compared with patients with the GG genotype.	0.53	0.999	35
				Drug responsive, N	47	9 (19.15%)	27 (57.45%)	11 (23.4%)	NR	NR	NR				
Haerian et al. [28]	2011	Chinese	Focal and generalized	Drug resistant, N	131	36 (27%)	72 (55%)	23 (18%)	NR	NR	NR	No significant association with ASM resistance.	0.18	0.784	288
				Drug responsive, N	146	35 (24%)	74 (51%)	37 (25%)	NR	NR	NR				
Subenthiran et al. [15]	2013	Malaysia	Focal	Drug resistant, N	182 * (162)	144 (75%)	13 (9.0%)	25 (16.0%)	NR	NR	NR	DRE patients were more likely to have the TT genotype (*p* < 0.001).	0.92	1.000	12
				Drug responsive, N	162 * (152)	59 (36.4%)	46 (28.4%)	57 (35.2%)	NR	NR	NR				
Subenthiran et al. [54]	2013	Malaysia	Focal	Drug resistant, N	162	56 (34.6%)	43 (26.5%)	63 (38.9%)	NR	NR	NR	DRE patients were more likely to carry the TT, while patients with the GG genotype were significantly more likely to respond to ASM (*p* < 0.001).	0.84	1.000	14
				Drug responsive, N	152	113 (74.3%)	12 (7.9%)	27 (17.8%)	NR	NR	NR				
Smolarz et al. [56]	2017	Poland	Not specified	Drug resistant, N	340	140 (41.0%)	68 (20.0%)	48 (14.0%)	36 (11.0%)	28 (8.0%)	20 (6.0%)	The GG genotype was significantly more frequent in DRE patients.	0.26	1.000	193
				Drug responsive, N	240	40 (16.7%)	62 (25.8%)	30 (12.5%)	58 (24.2%)	26 (10.8%)	24 (10%)				
Ajmi et al. [16]	2018	Tunisia	Focal and generalized	Drug resistant, N	46	15 (32.6%)	22 (47.8%)	9 (19.6%)	NR	NR	NR	DRE patients had significantly higher frequencies of the GT and TT genotypes compared to drug-responsive patients (*p* = 0.025).	0.48	1.000	42
				Drug responsive, N	107	58 (54.2%)	40 (37.4%)	9 (8.4%)	NR	NR	NR				
Zhao et al. [33]	2020	China	Focal and generalized	Drug resistant, N	117	15 (13%)	62 (53%)	23 (20%)	12 (10%)	5 (4%)	NR	The GG genotype frequency was significantly higher in ASM-responsive patients (*p* = 0.046).	0.30	0.979	132
				Drug responsive, N	128	29 (23%)	58 (45%)	23 (18%)	13 (10%)	5 (4%)	NR				
Maqbool et al. [13]	2021	Pakistan	Idiopathic generalized epilepsy	Drug resistant, N	110	4 (3.6%)	NR	16 (14.5%)	NR	51 (4.64%)	39 (35.5%)	The AA genotype was associated with ASM resistance compared to the TT wild-type genotype (*p* = 0.001).	0.64	1.000	32
				Drug responsive, N	127	5 (3.9%)	NR	47 (37.0%)	NR	42 (33.1%)	33 (26.0%)				
High statistical power															
Ufer et al. [40]	2009	Germany	Focal and generalized	Drug resistant, N	70	21 (30%)	37 (52.9%)	12 (17.1%)	NR	NR	NR	No significant association with ASM resistance.	0.25	0.843	155
				Drug responsive, N	102 * (103)	28 (27.45%)	47 (46.08%)	27 (26.47%)	NR	NR	NR				
Haerian et al. [28]	2011	Indian	Focal and generalized	Drug resistant, N	67	8 (12%)	33 (49%)	26 (39%)	NR	NR	NR	No significant association with ASM resistance.	0.26	0.850	142
				Drug responsive, N	93	18 (19%)	48 (52%)	27 (29%)	NR	NR	NR				
Balan et al. [50]	2013	India	Temporal with hippocampal sclerosis (MTLE-HS)	Drug resistant, N	256 * (259)	29 (11.3%)	129 (50.4%)	98 (38.3%)	NR	NR	NR	No significant association with ASM resistance.	0.15	0.848	404
				Drug responsive, N	199 * (201)	29 (14.6%)	86 (43.2%)	84 (42.2%)	NR	NR	NR				
Tamimi et al. [38]	2021	Jordan	Not specified	Drug resistant, N	46	19 (41.3%)	20 (43.5%)	5 (10.9%)	2 (4.3%)	NR	NR	DRE was 9 times more likely in females with the TT genotype compared to those with the CC genotype (*p* = 0.04).	0.37	0.843	78
				Drug responsive, N	40	11 (27.5%)	20 (50%)	8 (20%)	1 (2.5%)	NR	NR				
Moderate statistical power															
Kim et al. [45]	2006	Korea	Unspecified	Drug resistant, N	99	19 (19.2%)	33 (33.3%)	11 (11.1%)	22 (22.2%)	12 (12.1%)	2 (2.0%)	No significant association with ASM resistance.	0.24	0.754	232
				Drug responsive, N	107	17 (15.9%)	40 (37.4%)	9 (8.4%)	21 (19.6%)	15 (14.0%)	5 (4.7%)				
Dong et al. [41]	2011	China	Focal and generalized	Drug resistant, N	157	37 (23.5%)	56 (35.7%)	32 (20.4%)	19 (12.1%)	11 (7.0%)	2 (1.3%)	No significant association with ASM resistance.	0.17	0.702	428
				Drug responsive, N	193	49 (25.4%)	67 (34.7%)	38 (19.7%)	18 (9.3%)	20 (10.4%)	1 (0.5%)				
Low statistical power															
Grover et al. [30]	2010	India	Focal and generalized	Drug resistant, N	92 * (95)	10 (10.9%)	40 (43.5%)	35 (38.0%)	2 (2.2%)	4 (4.3%)	1 (1.1%)	No significant association with ASM resistance.	0.19	0.573	357
				Drug responsive, N	128 * (133)	12 (9.4%)	52 (40.6%)	58 (45.3%)	1 (0.8%)	5 (3.9%)	0 (0%)				
Sayyah et al. [11]	2011	Iran	Focal and generalized	Drug resistant, N	132	31 (23.5%)	60 (45.5%)	37 (28.0%)	2 (1.5%)	2 (1.5%)		No significant association with ASM resistance.	0.14	0.525	585
				Drug responsive, N	200	36 (18.0%)	97 (48.5%)	61 (30.5%)	2 (1%)	4 (2%)					
Seven et al. [49]	2014	Turkey	Unspecified	Drug resistant, N	69	17 (25%)	32 (46%)	17 (25%)	1 (1%)	2 (3%)		No significant association with ASM resistance.	0.25	0.687	193
				Drug responsive, N	83	20 (24%)	36 (43%)	24 (29%)	2 (3%)	1 (1%)					
Very low statistical power															
Hung et al. [12]	2007	Taiwan	Focal and generalized	Drug resistant, N	114	37 (32%)	58 (51%)	19 (17%)	NR	NR	NR	No significant association with ASM resistance.	0.09	0.289	1185
				Drug responsive, N	213	76 (36%)	100 (47%)	37 (17%)	NR	NR	NR				
Sánchez et al. [35]	2010	Spain/Caucasians	Focal and generalized	Drug resistant, N	111	48 (43.2%)	43 (38.7%)	20 (18.0%)	2 (1.6%)	3 (2.4%)	NR	DRE patients had a higher frequency of the GG genotype compared to the TT genotype (*p* = 0.03)	0.13	0.491	574
				Drug responsive, N	178	66 (37.1%)	74 (41.6%)	38 (21.3%)	NR	NR	NR				
Escalante-Santiago et al. [48]	2014	Brazil	Focal	Drug resistant, N	22	31.8% **	36.4% **	18.2% **	4.5% **	9.1% **	NR	No significant association with ASM resistance.	0.25	0.181	190
				Drug responsive, N	7	28.6% **	42.9% **	28.6% **	NR	NR	NR				
Zhou et al. [37]	2015	China	Focal and generalized	Drug resistant, N	153 * (156)	48 (31.4%)	74 (48.3%)	31 (20.3%)	NR	NR	NR	No significant association with ASM resistance.	0.07	0.196	2197
				Drug responsive, N	233 * (235)	68 (29.2%)	112 (48.1%)	53 (22.7%)	NR	NR	NR				
Xue & Lu [43]	2016	Chinese Han	Focal and generalized	Drug resistant, N	104	35 (33.65%)	53 (50.96%)	16 (15.38%)	NR	NR	NR	No significant association with ASM resistance.	0.06	0.125	2675
				Drug responsive, N	150	54 (36.00%)	72 (48.00%)	24 (16.00%)	NR	NR	NR				
Attia et al. [57]	2024	Egypt	Focal and generalized	Drug resistant, N	67	20 (29.9%)	34 (50.7%)	13 (19.4%)	NR	NR	NR	No significant association with ASM resistance.	0.1786186	0.4403930	302
				Drug responsive, N	67	24 (35.8%)	28 (41.8%)	15 (22.4%)	NR	NR	NR				

* The first number represents the calculated sum of the genotypes, whereas the numbers in parentheses correspond to the values provided in the reference article. ** Converted from a proportional to a percentage frequency.

**Table 3:** The rs1128503 (c.1236C>T, p.Gly412=) genotype frequencies, effect sizes, study power, and required sample size to achieve power (1 − β) = 0.8.

Author	Year	Population	Epilepsy Type	Drug Resistant, N	CC, N (%)	CT, N (%)	TT, N (%)	Drug Responsive, N	CC, N (%)	CT, N (%)	TT, N (%)	Conclusion	G-Power Calculations		
													Effect Size	Power 1 − β	Needed Sample Size
Very high statistical power															
Maleki et al. [58]	2010	Iran	Focal and generalized	132	24 (18.18%)	65 (49.24%)	43 (32.57%)	200	28 (14%)	87 (43.5%)	85 (42.5%)	In females, CC and CT genotypes were linked to a higher risk of DRE compared to TT (*p* = 0.02 and *p* = 0.008, respectively).	0.22	0.950	228
Sporis D et al. [22]	2011	Croatia	Focal	56	23 (41.1%)	21 (37.5%)	12 (21.4%)	43	10 (23.3%)	20 (46.5%)	13 (30.2%)	No significant association with ASM resistance.	0.37	0.916	72
Zhao et al. [33]	2020	China	Focal and generalized	117	13 (11%)	57 (49%)	47 (40%)	128	24 (19%)	54 (42%)	50 (39%)	No significant association with ASM resistance.	0.26	0.965	141
Maqbool et al. [13]	2021	Pakistan	Idiopathic generalized epilepsy	110	41 (37.3%)	52 (47.3%)	17 (15.5%)	127	35 (27.6%)	43 (33.9%)	49 (38.6%)	DRE patients had significantly higher frequencies of the CC and CT genotypes (*p* = 0.0003).	0.64	1.000	24
Zhu J et al. [59]	2023	China	Focal and generalized	61	5 (8.2%)	24 (39.3%)	32 (52.5%)	109	12 (11%)	61 (56%)	36 (33%)	DRE patients were more likely to carry the TT genotype (*p* = 0.013).	0.39	0.997	63
High statistical power															
Grover et al. [30]	2010	India	Focal and generalized	95	13 (14.1%)	49 (53.3%)	30 (32.6%)	129 * (133)	23 (17.8%)	54 (41.9%)	52 (40.3%)	No significant association with ASM resistance.	0.23	0.870	185
Haerian et al. [28]	2011	Chinese	Focal and generalized	131	27 (21%)	59 (45%)	45 (34%)	146	35 (24%)	74 (51%)	37 (25%)	No significant association with ASM resistance.	0.19	0.816	267
Moderate statistical power															
Kwan et al. [18]	2009	Han Chinese	Not specified	180 * (194)	19 (10.6%)	79 (43.9%)	82 (45.6%)	257 * (270)	34 (13.2%)	123 (47.9%)	100 (38.9%)	No significant association with ASM resistance.	0.14	0.759	481
Low statistical power															
Seo et al. [17]	2006	Japan	Focal and generalized	126	16 (12.7%)	49 (38.9%)	61 (48.4%)	84	15 (17.9%)	30 (35.7%)	39 (46.4%)	No significant association with ASM resistance.	0.16	0.520	390
Kim et al. [45]	2006	Korea	Not specified	97	18 (18.6%)	40 (41.2%)	39 (40.2%)	107	17 (15.9%)	54 (50.5%)	36 (33.6%)	No significant association with ASM resistance.	0.19	0.675	270
Hung et al. [12]	2007	Taiwan	Focal and generalized	114	12 (10%)	51 (45%)	51 (45%)	213	27 (13%)	104 (49%)	82 (38%)	No significant association with ASM resistance.	0.15	0.698	411
Vahab et al. [29]	2009	India	Not specified	112	30 (26.79%)	44 (39.29%)	38 (33.92%)	119	25 (21.01%)	46 (38.65%)	48 (40.34%)	No significant association with ASM resistance.	0.16	0.562	390
Ajmi et al. [16]	2018	Tunisia	Focal and generalized	46	14 (30.4%)	23 (50%)	9 (19.6%)	107	42 (39.3%)	48 (44.9%)	17 (15.9%)	No significant association with ASM resistance.	0.20	0.570	254
Very low statistical power															
Lakhan et al. [27]	2009	North India	Generalized and focal	94	12 (12.8%)	52 (55.3%)	30 (31.9%)	231	29 (12.6%)	124 (53.7%)	78 (33.8%)	No significant association with ASM resistance.	0.04	0.093	5787
Meng et al. [31]	2011	China	Focal and generalized	24	2 (8.3%)	10 (41.7%)	12 (50%)	60	5 (8.3%)	29 (48.3%)	26 (43.4%)	No significant association with ASM resistance.	0.14	0.189	503
Haerian et al. [28]	2011	Malays	Focal and generalized	125	24 (18%)	63 (46%)	38 (36%)	123	22 (18%)	51 (41%)	50 (41%)	No significant association with ASM resistance.	0.11	0.328	779
Haerian et al. [28]	2011	Indian	Focal and generalized	67	14 (21%)	31 (46%)	22 (33%)	93	20 (22%)	44 (47%)	29 (31%)	No significant association with ASM resistance.	0.04	0.074	5056
Dong et al. [41]	2011	China	Focal and generalized	157	22 (14.0%)	72 (45.9%)	63 (40.1%)	193	20 (10.4%)	92 (47.7%)	81 (41.9%)	No significant association with ASM resistance.	0.10	0.394	895
Balan et al. [50]	2013	India	Temporal with hippocampal sclerosis	259	32 (0.12)	110 (0.43)	117 (0.45)	200 * (201)	29 (14.5%)	78 (39%)	93 (46.5)	No significant association with ASM resistance.	0.08	0.445	1022
Escalante et al. [48]	2014	Brazil	Focal	22	6 (27%)	12 (55%)	4 (18%)	7	1 (14%)	4 (57%)	2 (29%)	No significant association with ASM resistance.	0.35	0.374	79
Tamimi et al. [38]	2021	Jordan	Not specified	46	17 (37%)	19 (41.3%)	10 (21.7%)	39 * (40)	12 (30.7%)	18 (46.2%)	9 (23.1%)	Females with the CC genotype were 18.7 times more likely to be observed in the DRE group compared to those with the TT genotype (*p* = 0.02).	0.13	0.177	553
Tang et al. [34]	2024	Vietnam	Focal and generalized	112	15 (13.4%)	54 (48.2%)	43 (38.4%)	101	15 (14.9%)	41 (40.6%)	45 (44.6%)	No significant association with ASM resistance.	0.15	0.287	413

* The first number represents the calculated sum of the genotypes, whereas the numbers in parentheses correspond to the values provided in the reference article.

**Table 4:** Summary of the genetic model meta-analyses for rs1045642 (c.3435C>T, p.Ile1145=) and the association with drug-resistant epilepsy (random-effects model).

Model	OR (95% CI)	Significant?	Heterogeneity (I^2^)
T vs. C (allele)	0.95 [0.87–1.05]	No	64%
Recessive (TT vs. TC + CC)	0.93 [0.81–1.08]	No	55%
Dominant (TT + TC vs. CC)	0.95 [0.83–1.09]	No	54%
Over-dominant (TC vs. TT + CC)	1.00 [0.92–1.09]	No	12%
TT vs. CC	0.92 [0.75–1.13]	No	64%
TT vs. TC	0.95 [0.83–1.08]	No	41%
TT vs. TC	0.97 [0.86–1.11]	No	40%

**Table 5:** Summary of the genetic model meta-analyses for rs2032582 (c.2677G>T/A, p.Ala893Ser/Thr) and the association with drug-resistant epilepsy (random-effects model).

Model	OR (95% CI)	Significant	Heterogeneity (I^2^)
T vs. C (allele)	0.98 [0.79–1.21]	No	87%
Recessive (TT vs. TC + CC)	1.01 [0.72–1.41]	No	87%
Dominant (TT + TC vs. CC)	0.99 [0.72–1.34]	No	85%
Over-dominant (TC vs. TT + CC)	0.99 [0.82–1.20]	No	67%
TT vs. CC	1.01 [0.72–1.41]	No	79%
TT vs. TC	1.02 [0.87–1.19]	No	27%
TT vs. TC	1.0 [0.75–1.34]	No	79%

**Table 6:** Summary of the genetic model meta-analyses for rs1128503 (c.1236C>T, p.Gly412=) and the association with drug-resistant epilepsy (random-effects model).

Model	OR (95% CI)	Significant	Heterogeneity (I^2^)
T vs. C (allele)	0.97 [0.87–1.09]	No	43%
Recessive (TT vs. TC + CC)	0.95 [0.81–1.13]	No	46%
Dominant (TT + TC vs. CC)	0.99 [0.85–1.16]	No	0%
Over-dominant (TC vs. TT + CC)	1.04 [0.92–1.17]	No	8%
TT vs. CC	0.96 [0.78–1.18]	No	30%
TT vs. TC	0.95 [0.80–1.12]	No	39%
TT vs. TC	1.05 [0.89–1.23]	No	0%

## Data Availability

No new data were created.
